# A Statistical Model to Identify Differentially Expressed Proteins in 2D PAGE Gels

**DOI:** 10.1371/journal.pcbi.1000509

**Published:** 2009-09-18

**Authors:** Steven H. Wu, Michael A. Black, Robyn A. North, Kelly R. Atkinson, Allen G. Rodrigo

**Affiliations:** 1 Bioinformatics Institute, University of Auckland, Auckland, New Zealand; 2 School of Biological Sciences, University of Auckland, Auckland, New Zealand; 3 Department of Biochemistry, University of Otago, Dunedin, New Zealand; 4 Department of Obstetrics and Gynaecology, University of Auckland, Auckland, New Zealand; Macquarie University, Australia

## Abstract

Two dimensional polyacrylamide gel electrophoresis (2D PAGE) is used to identify differentially expressed proteins and may be applied to biomarker discovery. A limitation of this approach is the inability to detect a protein when its concentration falls below the limit of detection. Consequently, differential expression of proteins may be missed when the level of a protein in the cases or controls is below the limit of detection for 2D PAGE. Standard statistical techniques have difficulty dealing with undetected proteins. To address this issue, we propose a mixture model that takes into account both detected and non-detected proteins. Non-detected proteins are classified either as (a) proteins that are not expressed in at least one replicate, or (b) proteins that are expressed but are below the limit of detection. We obtain maximum likelihood estimates of the parameters of the mixture model, including the group-specific probability of expression and mean expression intensities. Differentially expressed proteins can be detected by using a Likelihood Ratio Test (LRT). Our simulation results, using data generated from biological experiments, show that the likelihood model has higher statistical power than standard statistical approaches to detect differentially expressed proteins. An R package, Slider (Statistical Likelihood model for Identifying Differential Expression in R), is freely available at http://www.cebl.auckland.ac.nz/slider.php.

## Author Summary

Many researchers use two dimensional polyacrylamide gel electrophoresis (2D PAGE) to identify proteins with different concentrations under different conditions. Several statistical methods have been used to identify these proteins, ranging from standard statistical tests to complex image analysis. Most of these methods fail to address the limitation of this technology, which is that when the concentration of a protein is too low, 2D PAGE is unable to detect this particular protein. Standard methodologies implemented in most software packages ignore these proteins completely. We propose an alternative approach based on the likelihood framework, which takes into account when the concentration of protein is above the detection level and below the threshold. Our results show that this model allows us to identify more proteins with different concentration levels under different conditions than the standard statistical approaches.

## Introduction

Two-dimensional polyacrylamide gel electrophoresis (2D PAGE) [1] separates thousands of proteins within a sample by their isoelectric points (pI) in the first dimension and their molecular weights in the second dimension. Gels are scanned and spot detection performed using commercial or in-house software packages. These programs convert gel images into vectors of matched spot volumes and most analyses are subsequently performed on these data [2]. 2D PAGE may be used to identify proteins that differentiate or characterize certain patient groups or sample sets. For instance, by comparing specimens from patients with a specified disease to a control group, statistical differences in the levels of proteins can be determined to identify proteins associated with a disease state that may serve as diagnostic or prognostic biomarkers [3].

Several statistical tests have been applied to detect differences in protein expression. These include the use of classical Student's t-test, Analyses of Variance [2], principle component analysis and partial least squares analysis [4],[5]. A key disadvantage with these methods is their failure to adequately address the difficulties of dealing with non-expressed or undetected proteins in some or all subjects within a group [6],[7].

There are three broad reasons to explain why a given protein may not be detected in 2D PAGE experiments: (1) the lack of sensitivity of the experimental setup or software to detect the presence of an expressed protein, usually a consequence of some threshold of detectable concentration [8]; (2) the true absence or non-expression of a protein; and (3) software-induced error, when proteins are incorrectly designated as being absent [9]. Some researchers have developed methods that impute missing values from the existing data [6]. However, without knowing the true causes of these missing values, imputation may introduce additional errors to the dataset [5],[7]; in particular, by ignoring the possibility that a protein may not be expressed in a certain group of subjects, imputation may lead to an elevation in the numbers of false negatives.

The problem of missing values may be addressed through the incorporation of missing observations into a statistical model of the data. Under the principle of likelihood, estimates of parameters (such as the mean expression intensity or the probability of expression) may then be obtained by computing the probability of obtaining the observed data, given different values of these parameters. The best estimates are those that maximize this probability, which is also called the maximum likelihood. Wood and co-workers [10] first proposed a statistical method to compute the likelihood for expressed proteins which simultaneously takes missing data into account along with expression profiles. Their method does not distinguish the processes that may account for why a protein is undetected. This means that the probability associated with non-detection is a composite of the probabilities of protein non-expression or expression below the level of detection.

In our paper, a new likelihood model is proposed that extends the approach of Wood et al. and is specifically applicable to situations where subjects belong to either a Case group or a Control group, in keeping with a case-control experimental design. This extended model allows for non-detected proteins and classifies them into two categories: either (a) the protein truly is not expressed, or (b) the protein is expressed but the expression level is below the limit of detection. We show how our proposed new method performs under simulations and compare results with standard statistical approaches commonly applied to detect differences in protein expression between groups. We also present an example using a subset of spots from a Case-Control 2D PAGE experiment.

## Materials and Methods

### 

#### Development of a likelihood model

Our new likelihood was calculated using two statistical distributions to describe the data (i.e., a generalized mixture model). In our development of the likelihood model, we assumed the following:

1. For each subject in the case or control groups, a single 2D PAGE gel was run. The model can be extended to include multiple PAGE gels per person, but that extension is not described here.
2. For all 2D gels, image processing software matched the spots and, for each gel, calculated relative volumes for each spot by dividing the uncorrected volume of each spot by the sum of all spot volumes on that particular gel. The relative volumes for each gel were log_2_ transformed before further analysis. Calculation of relative spot volumes is roughly equivalent to mean subtraction on the log scale, and thus provides a simple approach to standardizing the distribution of spot volumes across gels, in a similar manner to the use of a fixed effects ANOVA model for the removal of linear array effects in microarray analysis [11]. In the data used here, the distributions of spot volumes across gels were very similar, resulting in only a minor correction. In cases where more serious inter-gel differences exist (e.g., differences in spread, or severe skewness after log transformation), more sophisticated approaches to standardization may be required.

For any individual for whom a 2D gel had been run, the probability that a given protein has a recorded volume depends on (1) the probability that the protein is expressed conditional on the group to which that subject belongs (modeled using the binomial distribution), and (2) the probability that the concentration of the protein is above the threshold limit of detection (modeled using a truncated and normalized normal distribution). The likelihood model is a mixture of these two probabilities.

The likelihood of obtaining the protein concentrations across all patients for each matched spot is the probability of obtaining these concentrations, given the parameters that determine the binomial and normal distributions (unique to each group), and the threshold level of detection. Each “spot” or set of matched protein intensities are treated as independent random variables, and analyzed separately. Let the parameters be collectively represented by 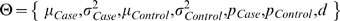 where 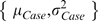 and 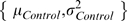 are the means and variances of the normal distributions of expressed protein concentrations for the Case and Control groups, respectively. Parameters 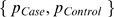 are the binomial probabilities that the protein is expressed in the case and control groups respectively, and *d* is the limit of detection.

Formally, we write the likelihood as
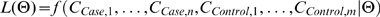
(1)


Where *f* is the likelihood function and 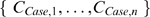 is the vector of concentrations in *n* subjects in the case group, and 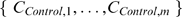 represent the m concentrations in the control group. For simplicity, in the following formulas, we will index the case and control groups as “1” and “2”, respectively.

We assume that the concentrations of proteins associated with each patient (conditional on their respective group parameters) are independent random variables. A proteomic gel scanner will scan image intensities at each coordinate of the gel. If the intensity is below the limit of detection, *d*, the scanner will typically leave the intensity value for that coordinate blank. The coordinates are then matched across the gels of different individuals. For our analyses, we include all coordinates where there is at least one (non-blank) value obtained for at least one individual (or gel). Consequently, in our model, we do not ignore all blank values, because across different individuals, some will have intensities above the limit of detection. When no concentration is recorded, *C_x,y_* is set to “NA” in our computer program, signaling that *C_x,y_*<*d*.

Consequently, we can rewrite Equation (1) as:
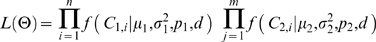
(2a)or as a log-likelihoods:
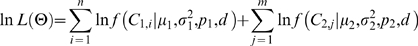
(2b)


Equations (2a and 2b) define the likelihood *L*(Θ), which represents the probability of obtaining the observed values of relative intensities, given hypothesized parameters Θ. For the *k*th subject of group *x*, we can partition the probability of obtaining the observed concentration, *C_x,k_*, conditional on *μ_x_*, *σ_x_^2^*, *p_x_* and *d* as:
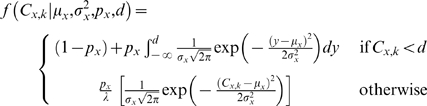
(3)and λ is the scaling factor to ensure the truncated normal distribution integrates to one:
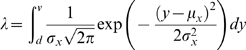
where *d* is the limit of detection and *ν* is the maximum expression value.

In Equation (3), The first term on the right hand side is the likelihood when the protein is not detected, and consists of two parts: the probability that the protein is not expressed, or the probability that the protein is expressed, but is below the limit of detection, *d*. The second term on the right-hand side is simply the ordinate of the truncated normal probability density function, and gives the likelihood when the protein has a detectable concentration. The truncated distribution is bounded between the limit of detection *d* and the maximum expression value *v*. The limit of detection *d* is assumed to be constant and known. The maximum expression value *v* is, of course, log_2_(100). Dividing by the scaling factor λ ensures that the truncated normal distribution integrates to one. The mean of the normal distribution *μ_x_* and the binomial probabilities *p_x_* are free parameters which can be estimated from the data in order to maximize the log-likelihood. The maximized log-likelihood allows us to identify differentially expressed proteins. We can test the null hypothesis that there is no difference between the mean expression intensities or the probabilities of expression between Case and Control using a Likelihood Ratio Test.

#### Application of the likelihood ratio test (LRT)

A protein is considered differentially expressed when a statistically significant difference between the mean expression intensities or the probability of expression of the two groups is detected. We use the LRT to compare two models to determine the difference between Cases and Controls.

We assume the variance of expression intensities is equal for both groups. The variance for each group is estimated separately then pooled according to the following formula.
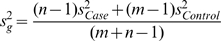
(4)


If the sample size for one group is too small (1 or less) and we are unable to estimated the variance for that group, then the empirical global variance is used for this particular group.

For the first simpler model, we assume the values for the parameters (mean expression intensities and the probability of expression) are common to both groups. Therefore there are only two free parameters in this simplified model and the log-likelihood is
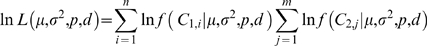
(5)


We also fit the more complicated model where these same parameters are allowed to have different values dependent upon the group (Equation 2b). The parameters that are allowed to vary between groups are referred to as free parameters. We let ln*L_1_* denote the maximum natural log of the likelihood from a model with more free parameters and ln*L_0_* be the maximum natural log-likelihood from the simpler model. The likelihood ratio statistic, Δ, is calculated as
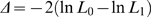
(6)


The null and alternative hypotheses for this test are
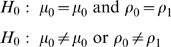



The maximum natural log-likelihoods from the two different models are calculated. The full model had four parameters which corresponded to mean expression intensities and probabilities of expression for both groups (Equation 2b). The null model only has one mean expression intensity and probability of expression, because it is assumed that these parameters are equal for both Cases and Controls.

When the sample size is large, the likelihood ratio statistic under the null hypothesis approaches a χ^2^ distribution with *n* degrees of freedom, where *n* is the difference in the number of free parameters between the null and alternative models. In the comparison between a single set of parameters for both Case and Control vs. separate parameters for Case and for Control, the difference in the number of free parameters (and, consequently, the degrees of freedom) is 2.

However, if the total number of individuals in the Case and Control groups is small (as in our 2D PAGE data), we may use a permutation procedure to generate the null distribution for the likelihood ratio statistic. For each protein, the normalized spot volumes are assigned randomly without replacement to patients, independent of case or controls status. This removes any effect due to the group membership of the individuals. This is done a large number of times (in our analyses, 1000 times), and for each permutation of the data, a likelihood ratio statistic is calculated. Combining the likelihood ratio statistics from these permutations generates a frequency distribution of the statistic under the null distribution, for which we are then able to determine the 95% quantile. A protein is considered statistically differentially expressed if the observed likelihood ratio statistic is greater than this quantile determined from the distribution.

In our analyses, the log-likelihood of each protein is estimated independently.

#### Simulation analysis

We determined the behavior of the likelihood-based approach using stimulated data and compared this with standard statistical methods such as Student's t-test. The simulated data were created based on real biological experimental results presented elsewhere. In this study [12], plasma samples were obtained from 24 women at 20 weeks of pregnancy; 12 of these women later developed preeclampsia and 12 remained healthy during pregnancy. Plasma was depleted of six high abundant proteins using the Multiple Affinity Removal System (Agilent Technologies). Images were created containing the protein spots on each gel, and spots were detected and matched using ImageMaster 2D Platinum software v6.01 (GE Healthcare). There were 803 spots matched across the gels. Data were then simulated in accordance with the experimental design using the summary statistics, mean expression intensities and global variances from this experiment.

We performed four simulations to generate four datasets, each corresponding to a different set of values for mean expression intensities and probabilities of expression. Based on the original data, simulated data were created by generating normalized percentage volumes for each protein in the “gel” for each of the 12 “subjects” in the case group and control group. For each gel, we simulated 1000 spots, drawing log-intensities from normal distributions centered on the mean log-intensities of case and control groups. The variance for the normal distribution was fixed at empirical global variance for all simulated dataset. The empirical global variance was calculated in two steps. Firstly, we pooled all the variances within each group to obtain the group variances for cases and controls, and then the global empirical variance was estimated by pooling these two variances (Equation 4).

The simulated datasets were generated according to the following four criteria:

#### Simulation 1. Different mean expression intensities with all spots expressed

The probabilities of expression were fixed at ‘1’ for both groups (i.e. all proteins expressed), but the groups had different mean expression intensities. The difference between the mean expression intensity in case and controls ranged from 0 to 2.5 standard deviations (SD) calculated from the global empirical variance. The limit of detection is ignored in this simulation because all values are expressed.

#### Simulation 2. Different probabilities of expression but the same mean expression intensities

In this simulation, proteins in the two groups had different probabilities of expression from 0.1–1, resulting in the number of expressed proteins on each gel being different in Cases and Controls. The mean expression intensities were identical for both groups (set to the empirical mean of −3.58 log2-volume units) and the limit of detection is set to negative infinity. For the Student's t-test, we applied one of two additional data pre-processing steps to handle missing values. Missing data were either ignored or replaced by a value equal to the lowest expression intensity obtained across all spots in all Cases and Controls.

#### Simulation 3. Different limit of detection and a fixed difference between the mean expression intensities

The limits of detection varied from 0% to 50% of the normal distribution of expression intensities, corresponding to the group with lower mean intensities. The probabilities of expression were fixed at ‘1’ for both groups, but if the simulated normalized percentage volume was below the limit of detection, then that protein was recorded as “non-expressed”. The mean log-intensities for the case and controls were fixed at −3.987 units and −3.174 units, respectively, equivalent to a difference of 1.25 SD units.

#### Simulation 4. Different mean expression intensities and same probability of expression between two groups

This is an extension of Simulation 1 and investigates the effect when not all spots are expressed. Both groups had the same probabilities of expression, but these now ranged from 0.1–1. The difference between the mean expression intensity in case and controls ranged from 0 to 2 SD. The limit of detection is set to empirical value (−8.67 log2-volume units), any simulated value below this threshold will be treated as missing data. Missing data were pre-processed for the Student's t-test as described for Simulation 2.

#### Application of model to simulated datasets

Differentially expressed proteins in each simulated dataset were identified using the LRT and Student's t-test using the software package R [13]. Likelihood optimization was performed using the Nelder and Mead algorithm [14]. To estimate the likelihood, we assume that the variance of expression intensities is equal for both groups. The variance for each group is estimated separately then pooled according to Equation (4). If the sample size for one group is too small (1 or less) and we are unable to estimated the variance for that group, then the empirical global variance are used for this particular group. For the Student's t-test, we assumed variances were unequal and corrected for degrees of freedom [15]. Proteins were classified as having significantly different levels of expression if p-values were less than 0.05. The power of each algorithm was determined by the proportion of simulations out of 1000 that were able to detect a given level of difference.

#### Application of model to 2D PAGE example

The 2D PAGE experiment described earlier consists of 803 matched spots per gel or sample. There were 12 samples from women who developed preeclampsia (Case group) and 12 from women who remained healthy during pregnancy (Control group). For each spot, the maximum likelihood was estimated under the two models and then the LRT was used to determine differentially expressed spots. The significance level of the hypothesis test was obtained by permuting the log-intensities across all patients 1000 times, reanalyzing the data under the null and alternative models, estimating the likelihood ratio for each permutation, and obtaining the value of the likelihood ratio that defined the 95% quantile of the distribution of likelihood ratios.

## Results

Our models were applied to the four simulated datasets.

### 

#### Simulation 1. Different mean expression intensities with all spots expressed

The proportion of proteins classified as differentially expressed between the two groups by the Student's t-test or LRT is summarized in Table 1. As expected, when all proteins are expressed, both methods demonstrated equivalent levels of power over the range of differences in mean expression intensities between groups tested.

**Table 1:** Results for different mean expression intensities between groups with all spots expressed.

Difference between means (SD)*	Case Mean	Control Mean	Student's t-test	LRT
0	−3.58	−3.58	3.8%	3.9%
0.25	−3.66	−3.50	10.4%	10.5%
0.5	−3.74	−3.42	20.1%	20.6%
0.75	−3.82	−3.34	40.5%	41.0%
1	−3.91	−3.26	64.4%	64.7%
1.25	−3.99	−3.17	82.9%	83.0%
1.5	−4.07	−3.09	93.7%	93.7%
1.75	−4.15	−3.01	98.2%	98.2%
2	−4.23	−2.93	99.7%	99.7%
2.25	−4.31	−2.85	100%	100%
2.5	−4.39	−2.77	100%	100%

Proportion of proteins classified as differentially expressed by each model.

*: Difference in mean expression intensities between cases and controls, expressed as proportions of the standard deviation, σ.

#### Simulation 2. Different probabilities of expression but the same mean expression intensities

The results of this simulation are presented in Table 2. When the probability of expression for case equals 0.2 and the probability of expression for control equals 1.0, the Student's t-test identified 4.0% of the differentially expressed spot if missing values are excluded, and 99.9% if all missing values are replaced by the global minimum. The LRT identified 85.9% from the same dataset.

**Table 2:** Results for equal mean expression intensities but the probability of expression differs between groups.

**A: Student's t-test, missing values excluded**											
Case: Probability of Expression											
		0.1	0.2	0.3	0.4	0.5	0.6	0.7	0.8	0.9	1
Control: Proability of Expression											
	0.1	0.4%									
	0.2	1.3%	2.4%								
	0.3	1.5%	2.9%	5.5%							
	0.4	2.5%	2.6%	4.4%	4.9%						
	0.5	1.6%	3.2%	3.7%	5.5%	5.5%					
	0.6	2.1%	2.8%	5.7%	4.7%	4.2%	4.1%				
	0.7	1.8%	4.1%	4.3%	6.2%	4.6%	5.0%	5.2%			
	0.8	2.4%	3.8%	5.9%	4.4%	4.2%	4.7%	5.3%	4.5%		
	0.9	1.1%	3.5%	3.8%	4.7%	3.7%	4.4%	5.7%	3.0%	4.8%	
	1	1.5%	4.0%	5.2%	5.4%	4.9%	5.2%	6.4%	5.2%	3.8%	5.7%
**B. Student's t-test, missing values replaced with global minimum**											
Control: Proability of Expression											
	0.1	1.6%									
	0.2	6.8%	4.5%								
	0.3	20.4%	7.6%	5.4%							
	0.4	39.0%	16.8%	7.9%	5.3%						
	0.5	58.1%	31.4%	16.9%	7.1%	4.1%					
	0.6	78.1%	53.6%	33.8%	17.3%	7.5%	5.1%				
	0.7	89.3%	71.2%	49.7%	32.1%	14.0%	5.7%	4.3%			
	0.8	96.6%	86.6%	74.0%	51.4%	29.8%	16.7%	7.4%	4.7%		
	0.9	99.8%	97.4%	88.4%	73.2%	55.3%	38.1%	22.2%	6.4%	3.2%	
	1	100.0%	99.9%	99.4%	95.4%	89.2%	70.4%	42.7%	20.8%	6.1%	6.2%
**C. Likelihood Ratio Test**											
Control: Proability of Expression											
	0.1	4.7%									
	0.2	4.1%	4.5%								
	0.3	3.9%	4.9%	6.4%							
	0.4	5.3%	4.3%	4.6%	5.3%						
	0.5	17.7%	6.6%	5.5%	5.2%	4.7%					
	0.6	25.3%	11.6%	7.2%	6.1%	4.4%	4.3%				
	0.7	37.7%	20.6%	9.7%	6.9%	4.9%	4.9%	5.4%			
	0.8	61.7%	30.6%	18.2%	10.2%	6.1%	6.7%	6.4%	4.2%		
	0.9	77.2%	47.4%	27.4%	15.3%	9.5%	6.9%	6.5%	2.8%	5.1%	
	1	97.8%	85.9%	52.4%	28.3%	14.3%	9.7%	8.3%	6.9%	4.7%	6.4%

Proportion of proteins classified as differentially expressed by each model.

When Student's t-tests were applied to datasets in which missing values were ignored, the majority of proteins were not classified as differentially expressed. This is the expected outcome, because the mean expression intensities of expressed proteins were identical in both groups and therefore the probability of successfully detecting differences is no greater than the value of α = 0.05. Consequently, a Student's t-test where missing values are ignored lacks the power to identify proteins with different expression probabilities between groups.

When missing values were assigned the global minimum log-intensity, the number of differentially expressed proteins detected by Student's t-test increased when the difference between probabilities of expression in the two groups increased. Substitution of missing values with the global minimum increased the power of the Student's t-test when the probability of expression was low for both groups because the estimated sample variance becomes very small. This is an artifact induced by replacing the many missing values by a constant, the global minimum.

When there are no differences between the probabilities of expression (diagonal in Table 2), the LRT returned the expected rate of 0.05 corresponding to the level of significance, but had lower power to detect differences between the groups. This is because the LRT does not substitute missing values; instead, the variance is estimated only on expressed values.

#### Simulation 3. Different limit of detection and a fixed difference between the two mean expression intensities

The difference between mean log-intensities for the Case and Control Groups were fixed at 1.25 SD units because in Simulation 1 this difference in mean intensities delivered >80% power (Table 1). When Student's t-tests were calculated ignoring non-expressed proteins, the statistical power dropped from 86% to 15% as the limit of detection increased, whereas the statistical power for the LRT dropped to 43.6% (Table 3). Again, replacement of missing values with some constant (in this case, the limit of detection) maintained the level of power of the Student's t-test at around 80%.

**Table 3:** Results for fixed difference in mean expression intensities and varying limits of detection.

Quantile on the normal distribution	Limits of detection	Student's t-test exclude missing data	Student's t-test global minimum for missing data	Likelihood Ratio Test
0%	-Infinity	86.3%	84.2%	84.4%
5%	−5.06	82.3%	83.9%	81.1%
10%	−4.82	74.1%	82.3%	74.6%
15%	−4.66	68.5%	82.2%	71.3%
20%	−4.53	61.6%	83.6%	69.7%
25%	−4.43	52.5%	82.4%	64.7%
30%	−4.33	45.6%	80.1%	59.2%
35%	−4.24	35.5%	82.1%	57.2%
40%	−4.15	31.3%	80.8%	56.2%
45%	−4.07	21.6%	78.7%	46.3%
50%	−3.99	15.4%	79.9%	43.6%

Proportion of proteins classified as differentially expressed by each model.

#### Simulation 4. Different mean expression and same probability of expression between two groups

Both groups had the same probabilities of expression, but these were no longer fixed at ‘1’. In contrast to the other simulations, replacement of missing values by the global minimum reduced the power of the Student's t-test to detect differences in expression intensities (Table 4). In contrast, the LRT and Student's t-test in which missing values were ignored performed equally well.

**Table 4:** Result for different mean expression intensities and same probability of expression between two groups.

Probability of Expression	M:0	M:0.25	M:0.5	M:0.75	M:1	M:1.25	M:1.5	M:1.75	M:2
**A. Student's t-test, missing values excluded**									
0.1	0.4%	0.9%	1.0%	1.4%	2.5%	4.1%	4.1%	5.4%	6.7%
0.2	2.5%	4.0%	5.7%	7.5%	12.5%	15.5%	24.2%	29.5%	35.0%
0.3	4.5%	4.4%	8.1%	15.4%	24.4%	30.0%	40.9%	52.9%	61.1%
0.4	4.1%	6.0%	10.1%	20.6%	31.0%	44.3%	57.0%	69.3%	80.0%
0.5	4.6%	5.9%	11.2%	23.4%	40.5%	53.1%	72.5%	82.0%	88.9%
0.6	5.7%	8.1%	13.8%	29.6%	46.8%	63.4%	79.4%	90.2%	94.5%
0.7	5.5%	8.7%	17.5%	33.4%	55.3%	73.5%	83.1%	94.5%	97.6%
0.8	5.5%	8.8%	20.9%	37.2%	60.1%	76.2%	91.8%	96.0%	99.1%
0.9	5.5%	9.4%	23.0%	41.0%	64.6%	83.0%	94.5%	98.1%	99.5%
1	5.9%	9.1%	20.8%	42.6%	70.5%	84.2%	95.9%	99.0%	99.9%
**B. Student's t-test, missing values replaced with global minimum**									
0.1	1.3%	1.7%	1.4%	1.6%	1.7%	1.6%	1.2%	2.0%	1.9%
0.2	4.6%	3.3%	4.9%	2.1%	4.0%	4.0%	3.4%	4.4%	4.6%
0.3	5.9%	4.5%	4.7%	3.5%	5.7%	6.1%	6.4%	5.6%	4.6%
0.4	5.2%	5.7%	4.7%	4.2%	5.0%	5.7%	5.8%	6.4%	7.8%
0.5	5.0%	4.9%	4.4%	6.6%	8.0%	7.4%	7.8%	8.7%	9.6%
0.6	5.5%	6.4%	5.3%	6.7%	6.2%	7.3%	7.8%	8.7%	9.3%
0.7	6.5%	5.4%	6.1%	7.1%	7.1%	10.0%	9.6%	11.2%	17.8%
0.8	4.8%	5.7%	6.3%	8.6%	11.7%	13.2%	17.3%	19.1%	24.7%
0.9	2.7%	4.7%	8.1%	12.3%	20.4%	25.4%	30.4%	35.8%	38.9%
1	5.4%	8.1%	19.1%	38.8%	65.4%	82.0%	94.5%	98.5%	99.8%
**C. Likelihood Ratio Test**									
0.1	5.2%	5.0%	5.9%	7.5%	7.2%	9.5%	10.4%	11.7%	15.0%
0.2	4.0%	5.7%	5.6%	9.0%	11.9%	14.2%	15.9%	23.9%	29.3%
0.3	5.2%	5.5%	8.9%	12.2%	18.9%	24.8%	32.6%	41.9%	47.6%
0.4	4.4%	6.0%	8.2%	16.3%	25.3%	35.7%	46.9%	56.8%	69.3%
0.5	4.1%	5.9%	12.1%	20.2%	34.1%	44.9%	61.8%	71.8%	81.1%
0.6	5.1%	7.8%	12.4%	25.7%	38.2%	55.2%	71.0%	83.0%	89.6%
0.7	4.9%	8.0%	15.8%	30.2%	47.1%	66.9%	76.7%	89.2%	94.0%
0.8	5.0%	9.4%	19.8%	33.2%	56.7%	71.1%	88.1%	92.0%	97.8%
0.9	5.8%	7.9%	22.4%	36.8%	60.4%	79.2%	90.7%	95.9%	98.8%
1	5.5%	8.4%	19.3%	39.3%	65.5%	82.3%	94.6%	98.6%	99.8%

Proportion of proteins classified as differentially expressed by each model.

#### Application of model to 2D PAGE data

**Figure 1:**
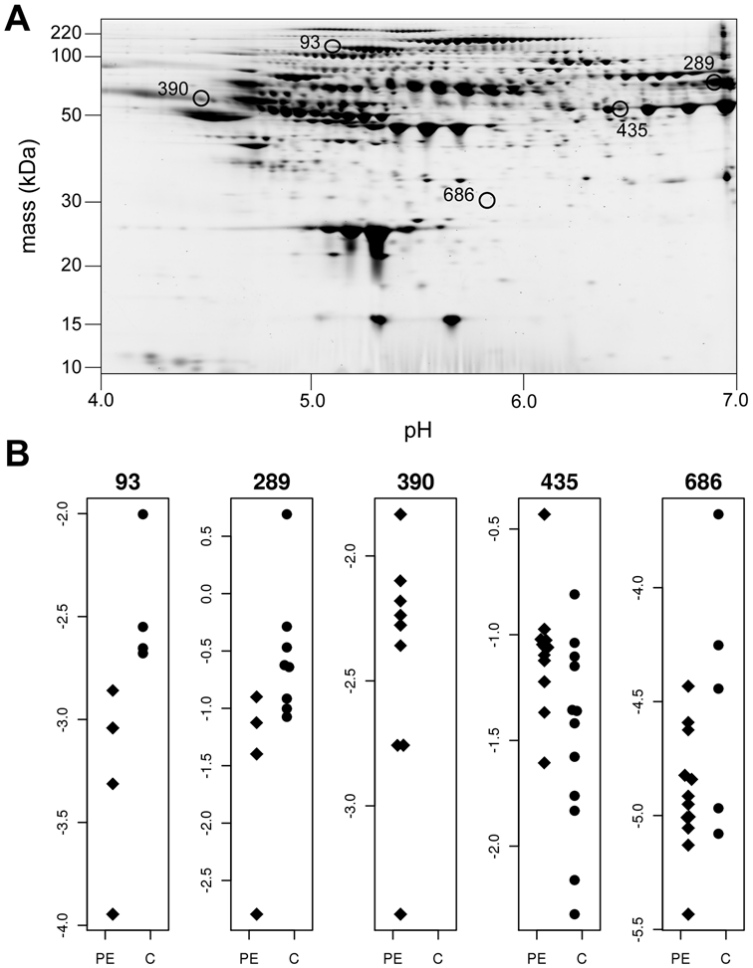
Five differentially expressed spots. (A) Five differentially expressed spots identified by the LRT on 2D PAGE. (B). Scatter plot of the five spots. PE = preeclampsia cases. C = Healthy controls.

The LRT identified 33 differentially expressed spots out of 803 match spots, of which five spots were selected exemplars (Figure 1). Each protein selected demonstrated different distributions in Cases and Controls. Spot 93 shows complete separation of Cases and Controls. In spot 289, the mean expression intensities and the number of expressed spots are different between groups, and spot 390 is only expressed in the Controls. Spot 435 has similar number of expressed spots but different mean expression intensities between two groups, whereas spot 686 has similar mean expression intensities but only five spots are expressed in the Case group and all 12 spots are expressed in controls. Table 5 shows the maximum likelihood derived from the two models, with the associated likelihood ratio statistic. As the likelihood ratio statistic was greater than the 95^th^ percentage percentile generated by 1000 permutations, we considered each of these protein spots to be differentially expressed. In contrast, when we applied a Student's t-test in which missing values are ignored, none of these proteins were statistically significant. The Student's t-test in which missing values are replaced by a global minimum was marginally better, identifying spots 289, 390 and 435 as significantly differentially expressed.

**Table 5:** Five differentially expressed spots identified by the Likelihood Ratio Test.

		Estimated Mean	Estimated Probability of expression	log maximum likelihood Null model	log maximum likelihood Alternative model	Likelihood Ratio Statistics	95th% quantile
Spot 93	Case	−3.29	0.33	−26.81	−10.46	16.41	11.58
	Control	−2.47	0.33		−8.15		
Spot 289.	Case	−1.55	0.33	−34.79	−13.21	15.11	12.49
	Control	−0.54	0.67		−14.02		
Spot 390	Case	−2.44	0.75	−21.47	−12.34	18.26	13.66
	Control	−2.48	0.00		−0.001		
Spot 435	Case	−1.09	1.00	−63.87	−5.90	41.37	27.86
	Control	−1.49	1.00		−37.28		
Spot 686	Case	−4.90	1.00	−26.64	−0.69	21.34	17.20
	Control	−4.48	0.42		−15.28		

## Discussion

In this paper, we developed a likelihood-based approach by using two statistical distributions to describe the data (i.e., a mixture model) to identifying proteins that are differentially expressed between two groups. True differential expression, under our definition, implies either a difference in the probabilities of expression between the two groups, or a difference in the mean expression levels, or both. Several standard statistical approaches only consider the difference in mean expression intensities. For any 2D PAGE experiments we should attempt to find the maximum number of truly differentially expressed spots and minimize both false positives and false negatives. The likelihood model classifies proteins that are undetected in some gels either as potentially expressed proteins that fall below the level of detection, or proteins that are not expressed. In so doing, the model tries to build a well-defined and biologically plausible picture of comparative protein expression. In contrast, standard statistical analyses (e.g. Student's t-tests) are forced to ignore “missing” proteins, or require some ad hoc pre-processing of data such as the replacement of missing values by a global constant or some other more sophisticated imputation process [6]. However, attempting to impute missing values when a protein is truly not expressed effectively increases the error. Inappropriate analytical methods can lead to loss of important information and potentially incorrect conclusions. For example, if a protein is expressed only in Cases, or only in Controls, application of standard statistical approaches may result in failure to recognize that the protein is a potential biomarker for that disease.

Our simulations highlight the contrast between the likelihood-based approach and the use of Student's t-tests. The performance of these approaches is summarized in Table 6. This table illustrates that LRT performs well under all four analyses, while the performance of Student's t-test varies between each analysis. In particular, when there are proteins that have not been identified in some gels, and are classed as “missing”, there are two kinds of t-test one may apply: one can choose to exclude “missing” values or one can replace these values with a global minimum. In two of our four sets of simulations, the Student's t-test in which missing values were replaced by a global constant had higher power than the LRT. This is because the estimated variance is artificially deflated as a consequence of replacing many expression intensities with the same constant. In contrast, the LRT performs better than the Student's t-test in Simulation 4, when the probabilities of protein expression are the same for the two groups, but the group-mean expression intensities differ. We expect that this situation, or one close to it, is more likely to mirror real experimental outcomes. Indeed, our application of the LRT on a small selection of proteins from a real biological experiment suggests that this is the case. We think that the likelihood model more realistically identifies the causes associated with “missing” data, and in so doing, provides a framework that is interpretable and intuitive.

**Table 6:** Compares the performance between four simulation analyses.

	Simulation 1	Simulation 2	Simulation 3	Simulation 4
Student's t-test, missing values excluded	Good	Low power	Low power	Good
Student's t-test, missing values replaced with global minimum	Not applicable	Good	Good	Low power
Likelihood Ratio Test	Good	Reasonable	Reasonable	Good

Mixture models are not new in the statistical literature and have been used in several other fields [16]. Similar models had been developed in other proteomic studies to handle missing values [17]; however, they have not been routinely applied to 2D PAGE experiments. The likelihood-based approach developed here can be applied to 2D PAGE experiments regardless of the physical or chemical system employed to generate the gel image and data. It also can be easily extended to allow multiple gels per patient and other, more complex, designs. What is required in these settings is to formulate appropriate distributional descriptions of the variances between gels within patients, and between patients within groups. In this regard, the process is no different from the parameterization under standard generalized linear mixture models. We are also developing extensions of this model for other proteomic data systems, including difference gel electrophoresis (DIGE) [18].

When we apply the same statistical test repeatedly, it is essential that multiple comparisons correction is applied after the analysis. Otherwise we are likely to discover large number of false positive differentially expressed proteins. In our analyses, we did not apply any correction for multiple tests, because our aim was to obtain estimates of the power and the false positive rates under different conditions. In practice, different multiple comparison procedures, such as the one proposed by Newton et. al. [19] can be implemented depending on the downstream analysis.
